# Auxiliary Diagnostic Signal for Deep-Level Detection

**DOI:** 10.3390/nano13212866

**Published:** 2023-10-29

**Authors:** Il-Ho Ahn, Dong Jin Lee, Deuk Young Kim

**Affiliations:** 1 Quantum-Functional Semiconductor Research Center, Dongguk University, Seoul 04620, Republic of Korea; jin514rin@naver.com (D.J.L.); dykim@dongguk.edu (D.Y.K.); 2 Division of Physics and Semiconductor Science, Dongguk University, Seoul 04620, Republic of Korea

**Keywords:** deep-level transient spectroscopy, I–V curve fitting

## Abstract

We propose and demonstrate that temperature-dependent curve-fitting error values of the Schottky diode I–V curve in the forward regime can be an auxiliary diagnostic signal as the temperature-scan Capacitance DLTS (CDLTS) signals and helps to work time-efficiently with high accuracy when using the Laplace Transform (LT)–DLTS or Isothermal Capacitance transient spectroscopy (ICTS) method. Using Be-doped GaAs showing overlapping DLTS signals, we verify that the LT–DLTS or ICTS analysis within a specific temperature range around the characteristic temperature $T_{peak}$ coincides well with the results of the CDLTS and Fourier Transform DLTS performed within the whole temperature range. In particular, we found that the LT–DLTS signals appeared intensively around $T_{peak}$, and we confirmed it with the ICTS result. The occurrence of the curve fitting error signal is attributed to the relatively increased misfit error by the increased thermal emission from the deep-level trap in the case near the $T_{peak}$, because the applied transport model excludes defect characteristics.

## 1. Introduction

Defects in semiconductor devices can have significant effects on their electrical transport properties. Among the traditional methods for electrical analysis of these defect characteristics, Capacitance Deep Level Transient Spectroscopy (CDLTS) [1] is the best known. The principle of the CDLTS capturing or emitting charge carriers at deep levels has also been adopted in many ways to Optical DLTS [2,3,4,5], Photoinduced Current Transient Spectroscopy (PICTS) [6,7,8,9,10], Fourier Transform (FT)–DLTS [11,12,13], Laplace Transform (LT)–DLTS [14,15,16], Charge based (Q)–DLTS [17,18,19,20], etc., according to the measurement environment and sample conditions. However, the analysis methods mentioned above are typically performed over a very wide temperature range (e.g., (60–400) K) using very short temperature intervals (e.g., 1 K) over very long periods of time (e.g., several hours in some cases, and over 12 h in specific cases). Isothermal DLTS (or Isothermal Capacitance Transient Spectroscopy (ICTS)) [21,22], which enables DLTS analysis at a specific temperature, also requires the temperature-scan DLTS first when the measurement sample is a complex system, additionally performed at the temperature specially selected among the overlapped DLTS signal [23]. Ultimately, most of the DLTS experiments for the entire temperature range are inevitable. Therefore, despite being good defect analysis tools with sensitive resolution, the long measurement time they all require has recently served as an obstacle to their widespread usage in industrial applications.

The present study addresses a methodology that a diagnostic signal similar to the DLTS signal can be obtained from curve fitting in the forward regime of the Schottky diode, and by using this diagnostic signal, the DLTS measurement and analysis time could be shortened in particular cases through LT–DLTS or ICTS measurement, without performing temperature-scan DLTS. We chose the simplest GaAs PIN solar cell structure [24] to demonstrate our methodology. GaAs is one of the representative III–V materials, and its DLTS study has been widely reported [25,26]. We fabricated a lateral Schottky diode to examine only the p-GaAs layer (100 nm) using DLTS with our new auxiliary diagnostic signal. The merit of selecting this lateral device structure is that deep-level analysis is possible by utilizing lateral transport characteristics even if the thickness of the channel is in the nanometer scale. We first show that the deep-level activation energy values in our sample obtained from conventional analysis by performing CDLTS measurements are similar to those previously reported by other groups. This means that the conventional DLTS measurement and interpretation of our sample are highly accurate. Meanwhile, by using capacitance transient data obtained experimentally for CDLTS analysis, other analysis methods, FT–DLTS and LT–DLTS analysis, are added to show that all three analysis methods provide similar results, proving that our FT–DLTS and LT–DLTS analysis methods are reliable. In this process, we found that the LT–DLTS or ICTS analysis method is the one among our analysis methods that can give a similar result by measuring around the $T_{peak}$. Generally, in LT–DLTS analysis, it is also a common analysis method to analyze all capacitance transient data obtained over a wide temperature range; but in the case where overlapped DLTS signals exist, such as the sample used in this study, it was found that similar results can be obtained, even by analysis in a small temperature range centered on the $T_{peak}$ of the peak with the highest intensity of DLTS signal. Therefore, our study suggests that when the LT–DLTS analysis method is used as an analysis method to obtain the deep level, the deep level corresponding to the $T_{peak}$ can be obtained even by simply measuring around the temperature range where the DLTS signal occurs. In other words, when a discrete DLTS signal exists, one deep level can be obtained from measurement and analysis around the characteristic temperature, but where overlapped DLTS signals exist, multiple deep levels can be obtained around the $T_{peak}$. In the case of this study, the samples used overlapped with the DLTS signals, and efficient deep-level analysis was possible with LT–DLTS analysis in a very small temperature range. We also double-validated LT–DLTS with ICTS at a specific temperature around $T_{peak}$.

On the other hand, we additionally proposed a method to estimate the $T_{peak}$ where the DLTS-like signal exists without a DLTS scan for the entire temperature range. That is the I–V curve fitting error signal as an auxiliary diagnostic signal. This technique uses the error generated in the process of fitting the I–V curve in the forward region of the Schottky diode by applying the transport model [27]. Since deep-level information is not included in the transport model, the experimental current values at the $T_{peak}$ at which the trapped carriers present in the deep level are emitted exhibit abnormal transport characteristics, and significant errors occur due to the difference between the experimental and theoretical current values. We show that this error signal is very similar to the DLTS signal. Therefore, if the error signal for the temperature is obtained in advance before the DLTS experiment, the $T_{peak}$ can be estimated from the error signal; then the LT–DLTS or ICTS signal can be obtained time-efficiently by measuring the vicinity of the $T_{peak}$. This study suggests that the LT–DLTS or ICTS analysis method using the I–V curve fitting error signal can obtain similar results, while reducing the measurement time, compared to the existing capacitance DLTS method.

The novelty of our work is that we utilized the fitting error of the Schottky diode I–V curve not as a by-product but as a key role for diagnosing the characteristic peak position and the number of deep levels of DLTS analysis. Finally, we also compared our method with other methods like TSC [28,29,30] (or TSCAP [30,31,32]), which might be considered auxiliary tools for DLTS. It is thought that the methodology of this study will help to utilize DLTS more efficiently in the industrial environment.

## 2. Sample and Experimental Scheme

**Figure 1:**
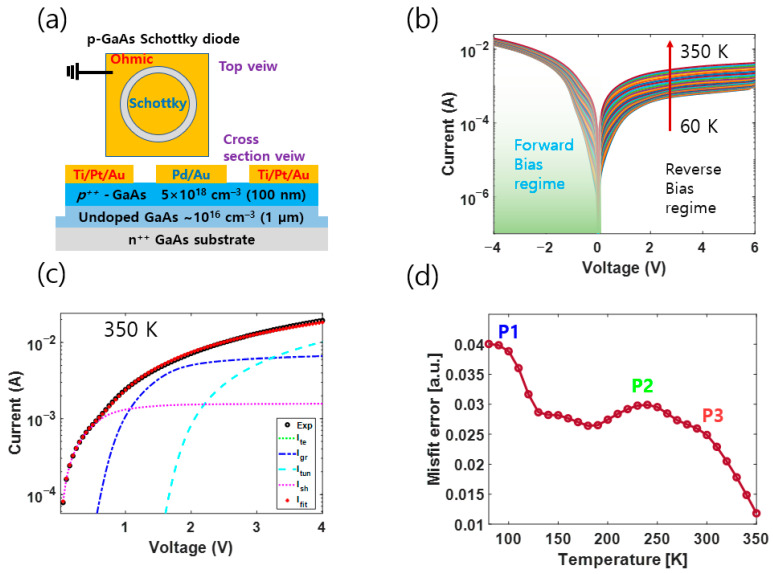
(**a**) Schematic structure of the p–GaAs Schottky diode. (**b**) I–V–T experimental data measured by temperature increment (∆T=10 K). (**c**) Curve fitting analysis at 350 K. (**d**) Error signal of I–V curve fitting.

**Figure 2:**
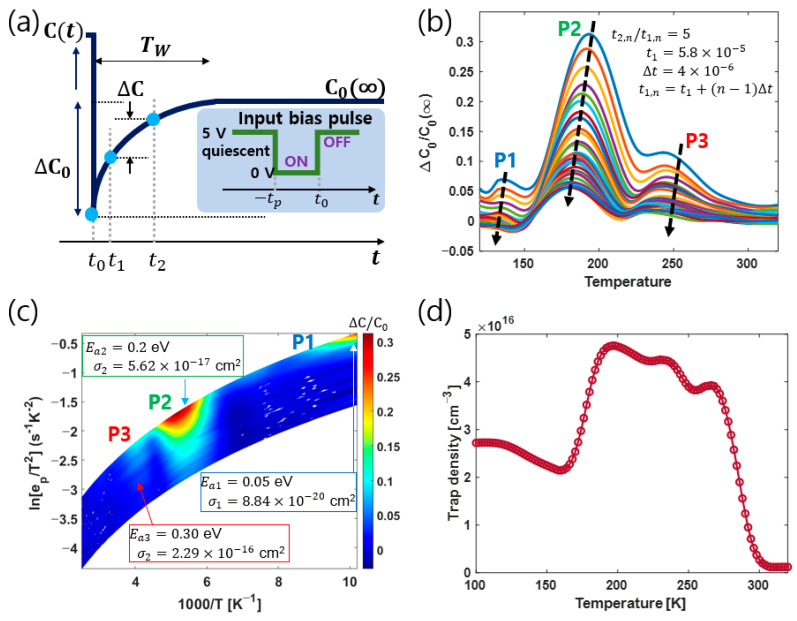
(**a**) Schematic of the CDLTS measurement. Period width $T_{W}$ was used in the FT–DLTS analysis in Figure 3. (**b**) Capacitance DLTS signals with $t_{w}$ variation as $t_{2}/t_{1}$ = 5. For the plot, n of $t_{1,n}$ was used from (2 to 500), increasing by 5. (**c**) Arrhenius contour plot for activation energy and to capture the cross-section. (**d**) Plot of trap density vs. temperature. Figure S5a of the SI shows the Capacitance transient data.

The test sample used in this study is a 100 nm thick Be-doped p-type GaAs epitaxial layer grown using the MBE method, supplied by iSensIRs [33] (Figure 1a). Detailed material characterizations of the Be-doped p-type GaAs epitaxial layer are given in the Supplementary Information (SI) [see Figures S1–S3]. A Schottky diode was fabricated for the I–V, CDLTS, FT–DLTS, and LT–DLTS analyses. As shown in Figure 1a, a circular Pd/Au (20 nm/100 nm) Schottky electrode (D ~ 500 μm) was formed on the p–GaAs cap layer. An ohmic contact metal of Ti/Pt/Au (20 nm/50 nm/100 nm) was formed on the p–GaAs cap layer as a ring electrode near the circular Schottky contact (Figure 2a). After the Schottky diode structure was fabricated, the I–V was measured using a Keithley 2614B system over the temperature range (80–400) K at 10 K intervals. For transport mechanism analysis in the forward bias region, measurements were taken at 25 mV intervals up to (0–1) V and then at 250 mV intervals up to (1–6) V. The C–DLTS measurements were performed using a Boonton 7200 and NI USB6363. The curve fitting model in Ref. [27] was used, along with the gradient descent and Newton methods. The FT–DLTS analysis was carried out using the methodology in Ref. [11]. One of the numerical inverse Laplace transform algorithms, the CONTIN algorithm in Ref. [34], was used for LT–DLTS analysis.

## 3. Results and Discussion

### 3.1. Error Signal from I–V Curve Fitting of the Schottky Diode

As mentioned in the introduction, if the $T_{peak}$ at which the DLTS signal appears can be known in advance, the measurement time can be drastically reduced by performing the LT–DLTS measurement only around that temperature. We propose an error signal through I–V curve fitting to determine this $T_{peak}$ without DLTS measurement for the entire temperature range.

Figure 1a shows the structure of the p–GaAs sample used in this study, along with a schematic of the Schottky diode. As the characteristics of the Schottky diode for conventional DLTS measurement, we checked the built-in potential 0.11 V at 130 K and the absolute reverse leakage current 0.85 mA at −5 V [see Figure S4a of the SI]. Although it shows a somewhat leaky characteristic, this means that there is no problem with DLTS measurement. The activation energy using reverse leakage current was about 0.06 eV, which was also confirmed to appear as one of the deep levels obtained through DLTS measurement [see Figure S4b of the SI]. This means that our Schottky diode is suitable for DLTS analysis (see the SI).

**Figure 3:**
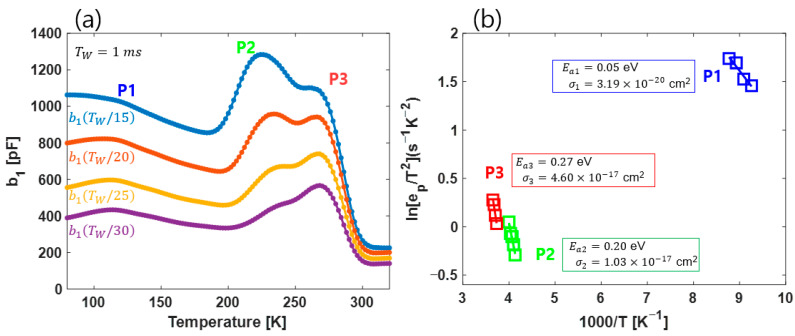
(**a**) $b_{1}$ plot of FT–DLTS. (**b**) Arrhenius plot of FT–DLTS. Plot points were obtained by only using $\tau (a_{1}(T_{W}/15),b_{1}(T_{W}/15))$ value.

Figure 1b shows the I–V curve measured by raising the temperature at 10 K intervals from (130 to 350) K. Figure 1c shows the result of curve fitting at 350 K using the transport model in the forward bias area of I–V data. The transport model that was used for IVT fitting was from Ref. [27], while the gradient descent and Newton methods were used for the fitting algorithm according to their routine. Table S1 of the SI summarizes the detailed transport model equations in Figure 1c. The misfit error at a given temperature was defined as follows:

(1) $$Misfit\;error=\frac{1}{N}\sum_{k=1}^{N}\left|Log\frac{I_{fitted,k^{th}point}}{I_{actual,k^{th}point}}\right|\;$$

where N is the number of points measured in the forward regime in Figure 1c. Figure 1d shows the misfit errors at all temperatures. Hereafter, we will call these misfit errors in Figure 1d error signals. Later, we will show that this error signal is very similar to the DLTS signal (see Figure 2a). The error signal in Figure 1d shows that error peaks from I–V curve fitting are formed around the characteristic temperatures $T_{peak,error}$ of (100, 250, and 280) K. This error peak can be considered to correspond to the DLTS peak at the characteristic temperature $T_{peak,DLTS}$ of the DLTS signal, i.e., $T_{peak,error}\;\approx \;T_{peak,DLTS\;}\equiv \;T_{peak}$. We could assume that this is because the error signal shape is similar to that of the DLTS peak: While the temperature increases, the charged carriers captured near the deep level are activated and emitted by thermal energy, and the excess current value increases. Since this phenomenon is not applied to the I–V transport model for IV curve fitting, it is inevitable that the misfit error relatively increases. The difference between the full width at half maximum (and peak position) of Figure 1d and the DLTS signal can be attributed to a problem of fitting accuracy in the process of obtaining the error signal. The more precise the curve fitting process is, the more accurate the error signal is comparable to the actual DLTS signal: Figure 1d shows three main peaks (P1, P2, P3), which correspond to the temperature positions of the $T_{peak}$ in the DLTS signal [see Figure 2b and Figure 3a]. However, the peak heights appear non-proportional. This discrepancy can be attributed to the relatively higher curve fitting error at low temperatures than at high temperatures.

The original purpose of the I–V curve fitting is to understand the current transport mechanism. However, our study does not suggest a new model for optimizing these fittings because our goal is to select a temperature range with a relatively large error and use it for the LT–DLTS analysis introduced in Section 4. Therefore, there is no need to conduct fitting for all temperatures. In other words, the purpose is not to obtain a strict $T_{peak}$ value but to determine the minimum measurable temperature range of DLTS based on the presence or absence of an error signal and $T_{peak}$. Therefore, the I–V–T measurement was performed at 10 K intervals, which is ten times the minimum interval (for example, △K=1) used for DLTS measurement. However, at each temperature, the fitting itself should use a rigorous algorithm to reduce errors to a minimum.

On the other hand, we also tried to use an additional method that is similar to the thermally stimulated current (TSC) signal to infer the characteristic temperature $T_{peak,DLTS}$ of the DLTS signal; i.e., we plotted the values at a specific bias voltage against the temperature in the I–V–T results of Figure 1b, but in our sample, the TSC-like signal was not found, which made it difficult to define $T_{peak}$ (data not shown here). To this point, we showed that the deep-level characteristic temperature $T_{peak,DLTS}$ can be inferred through the $T_{peak,error}$ obtained from the error signal from the I–V curve fitting method immediately after measuring the I–V characteristics. Before revealing how we applied our methodology to LT–DLTS analysis, first, for comparison with the conventional defect analysis method, CDLTS and FT–DLTS analysis results were obtained as references.

### 3.2. Activation Energy from Capacitance DLTS

Figure 2a shows a schematic of the capacitance transient process after the pulse edge time $t_{0}$ to explain the parameters of the formula used in the CDLTS and FTDLTS analyses. The filling pulse time $t_{p}=50$ ms and $T_{W}=0.1$ ms were used. Figure 2b presents a normalized capacitance DLTS signal according to the change of rate window ($t_{w}=\frac{1}{e_{p}}=(t_{2}-t_{1})ln(t_{2}/t_{1}$)) with time ratio $(t_{2}/t_{1}$=5). Figure 2c shows the Arrhenius contour plot expressed using Figure 2b. The formula used here is from Ref. [35]:

(2) $$ln\left(\frac{e_{p}}{T^{2}}\right)=ln\left(\frac{\sqrt{6}\pi 1.5k^{2}m_{h}^{*}\sigma_{h}}{h^{3}}\right)+\left(\frac{-△E_{a}}{1000k}\right)\frac{1000}{T}$$

where $e_{p}$ is the hole emission rate, T is the absolute temperature, k is Boltzmann’s constant, $m_{h}^{*}$ is the hole effective mass, and h is Plank’s constant. Figure 2c shows the activation energy $E_{a}$ and captured cross-section for the three peaks (P1, P2, P3), while Figure 2b correspondingly expresses the temperature positions. Figure 2d shows the trap density for each temperature. For trap density $N_{T}$, the following formula was used [35]:

(3) $$N_{T}\approx 2N_{A}\frac{△C_{0}}{C_{0}(\infty )}$$

Here, $N_{A}$ should be strictly used as the value calculated from the C–V measured at each temperature. However, for simplicity, we used $N_{A}$ obtained at 300 K to calculate $N_{T}$ at all temperatures. We did this because our purpose is to find the number of deep levels and the activation energy from the Arrhenius plot in the minimum experimental temperature range, so we omitted precise measurement of the value of $N_{A}$ of $T_{peak}$ and/or $N_{A}$ of all temperatures. The FT–DLTS and LT–DLTS methods, which are later comparatively analyzed, also have formulae for obtaining trap densities. However, since our current interest is not in those formulae, we introduce the trap density obtained only from CDLTS. Moreover, from now, the confirmation and comparison of the $E_{a}$ for the three peaks observed from FT–DLTS and LT–DLTS show that they are similar to those from CDLTS; therefore, the advantage of LT–DLTS will be demonstrated, in that it is capable of effective measurement time.

Figure 2b,c find that P1 exists below 150 K, P2 exists around 200 K, and P3 exists around 250 K. The existence of these peaks was noticed because we performed experiments at 2 K intervals from (80 to 400) K. To obtain one capacitance transient data, the temperature ramping rate was 1 K/10 min, and the experiment time to obtain all data was about 27 h. We could proceed faster if we were to adjust the temperature ramping rate, but conducting precise experiments might take longer. The $E_{a}$ values for the three peaks we analyzed were confirmed to be very similar to the $E_{a}$ values for Be-doped GaAs that have previously been published by other groups [36], where $E_{a1}$, $E_{a3}$ are related to the arsenic vacancy–interstitial pair and $E_{a2}$ is speculated as a complex type of defect.

As mentioned above, when conducting CDLTS analysis, measurements have to be taken for a long time over a wide temperature range. Therefore, in most cases, when performing a DLTS experiment, the first step is to roughly determine the measurement temperature range and measurement temperature interval and use a fast temperature ramping rate to obtain a thermally stimulated capacitance signal. Another common method is for precise experiments to be performed again after quickly testing the DLTS signal using only one measurement variable, $V_{p}$ or $t_{p}$. As such, it is often necessary to conduct a separate pre-test to determine whether the deep level is expressed, and how large a temperature range should be selected. We can additionally reduce the experiment time since we can accomplish this with only a basic I–V–T test.

### 3.3. Activation Energy from Fourier Transform–DLTS

In the next step, we will consider deep levels obtained from the FT–DLTS method, one of the most common defect analysis methods. We note that this analysis method also uses the same capacitance transient data, so the measurement time required to obtain the characteristic peaks is inevitably the same as that of CDLTS. Although there are advantages of analysis using FT–DLT, it is also difficult to expect a dramatic reduction in measurement time. In the FT–DLTS analysis, the capacitance transient is interpreted as a Fourier series, while the DLTS signal can be expressed as a Fourier coefficient and is expressed as follows [11]:

(4) $$a_{n}\left(\tau \right)=\frac{2△C}{T_{W}}\exp\left(-\frac{t_{0}}{\tau }\right)[1-exp(-T_{W}/\tau )]\frac{1/\tau }{\frac{1}{\tau^{2}}+n^{2}\omega^{2}}$$

(5) $$b_{n}\left(\tau \right)=\frac{2△C}{T_{W}}\exp\left(-\frac{t_{0}}{\tau }\right)[1-exp(-T_{W}/\tau )]\frac{n\omega }{\frac{1}{\tau^{2}}+n^{2}\omega^{2}}$$

where $T_{W}=1$ ms, $t_{p}=50$ ms, $\omega =2\pi /T_{W}$, and ΔC is the amplitude of the capacitance transient. The emission rate $e_{p}$ can also be determined from the ratio of Fourier coefficients, as follows:

(6) $${1/e}_{p}\left(a_{n},a_{k}\right)=\frac{1}{\omega }\sqrt{\frac{a_{n}-a_{k}}{k^{2}a_{k}-n^{2}a_{n}}}$$

(7) $${1/e}_{p}\left(b_{n},b_{k}\right)=\frac{1}{\omega }\sqrt{\frac{{kb}_{n}-nb_{k}}{k^{2}nb_{k}-n^{2}kb_{n}}}$$

(8) $${1/e}_{p}\left(a_{n},b_{n}\right)=\frac{1}{n\omega }\frac{b_{n}}{a_{n}}$$

Figure 3a shows the $b_{1}$ signal for the $T_{W}$ change. Using the relational expression for $a_{n}$, $b_{n}$, and $e_{p}$, the Arrhenius plot with $e_{p}\left(a_{1},b_{1}\right)$ values was created and is shown in Figure 3b. The $E_{a}$ values of the three peaks in Figure 3a are shown in Figure 3b. It can be seen that these values are very close to the $E_{a}$ values shown in Figure 2c. It can therefore be seen that the deep levels of the p–GaAs sample shown in our experiment form three main peaks, and that the activation energy is almost identical to the reported value. Finally, to verify our proposal, in the next section, we demonstrate that the same effect that is achieved using CDLTS and FT–DLTS can be confirmed in a very short temperature range by using the LT–DLTS analysis method.

### 3.4. Minimal Test Time Using the Laplace Transform–DLTS

The LT–DLTS analysis method is also widely used for deep-level analysis along with the FT–DLTS. In the LT–DLTS, capacitance transient data C(t) can be expressed as the Laplace transform of the spectral density function $f(e_{p})$, as follows [34]:

(9) $$C(t)=\int_{0}^{\infty }f\left(e_{p}\right)exp(-e_{p}t)de_{p}$$

where $e_{p}$ is the emission rate, and t is time. The same capacitance transient data obtained in the CDLTS experiment was used in the LT–DLTS analysis. The LT–DLTS analysis assumes that the capacitance transient data C(t) consists of a superposition of charged carriers with individual decay times of carriers. Therefore, the $f(e_{p})$ can be obtained by numerically performing the inverse Laplace transform of the C(t) obtained through the experiment. This $f(e_{p})$ is called the LT–DLTS signal. By decomposing overlapped information, it is possible to predict the number of traps (the number of deep levels). Since the same transient data obtained in C–DLTS analysis is used, LT–DLTS analysis for all temperatures is performed, and the Arrhenius plot is performed using the peak position of the LT–DLTS signal to obtain the deep-level activation energy and capture the cross-section.

**Figure 4:**
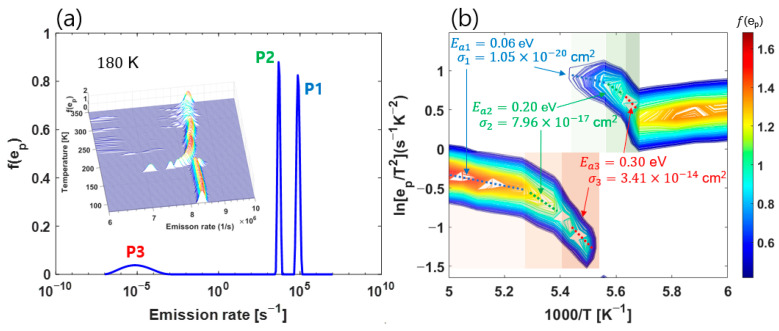
(**a**) Spectral function $f(e_{p}$) obtained from the Inverse Laplace transform of C(t) at 180 K. The whole data is shown in the inset. (**b**) Arrhenius contour plot of the LT–DLTS.

If the deep level has energy bandwidth or if several deep levels overlap, the CDLTS signal will appear broad. The LT–DLTS analysis appears to be capable of more precise decomposition than CDLTS, even under these circumstances [30]. As shown in Figure 2b and Figure 3a, in the special case where three DLTS signals overlap, we want to show that if the LT–DLTS analysis method is applied around the $T_{peak}$ ((190–200) K) where all DLTS signals overlap, the same analysis results as the CDLTS analysis obtained in all temperature ranges can be obtained. We used open Python code to obtain $f(e_{p})$ [34,37]. Figure 4a shows $f(e_{p})$ at 180 K. Figure 4b shows an Arrhenius contour plot created using all $f(e_{p})$ data obtained from the Inverse Laplace transform, as was done in Figure 4a for all measured temperatures. In the Arrhenius plot using the $f(e_{p})$ peak of LT–DLTS, we must focus on the region with a negative slope. This is because doing so can allow $E_{a}$ to be obtained and capture cross-section values through the slope and the y-axis intercept, respectively.

At all temperatures, three peaks appear, as shown in Figure 4a, but Figure 4b shows only more than 50% of the maximum intensity of $f(e_{p})$ as a contour plot. The data are presented like this to allow for the two main peaks to be observed in further detail, since—in our data—the main peak is most prominently decomposed into two peaks in the range (175–200) K. The LT–DLTS analysis, as shown in Figure 2b and Figure 3a, indicates that the separation was clearest near $T_{peak}\approx 200$ K.

The P3 peak values in Figure 4a were excluded from the contour plot for analysis due to the peak intensity being very small and the peak positions not regularly changing with temperature. However, it was possible to obtain all the deep-level information simply by observing the changes of the two main peaks, P1 and P2, decomposed at around 200 K. The two peaks separated in Figure 4b can be regarded as forming two groups for convenience. Let us refer to these as the upper group and the lower group.

Looking at the upper group, all three slopes representing the different $E_{a}$ values are clearly along the temperature range (5.45 to 5.65) in 1000/K values (x-axis). Even in the lower group, three slopes are distinguished in 1000/K values between about (5 and 5.47). In other words, in Figure 4b, both peaks P1 and P2 could show three slopes with temperature change. We also notice that even if the 1000/K value is further narrowed to the range (5.4–5.6), three distinct slopes are evident at once through the upper and lower groups.

To summarize our results, the Arrhenius plot of LT–DLTS shows that (1) several discrete deep levels can be identified along the specific temperatures using one spectral peak, and/or (2) different deep levels can be identified within the short specific temperature ranges using several peaks. From the above property (1), we can guess that LT–DLTS can repeatedly express the spectral peak’s slope in an arbitrary temperature range. This is considered an advantage of LT–DLTS analysis. In actuality, Figure 4b shows that the activation energy values obtained by linear fitting along the dotted line show the same results at different locations. This result is also consistent with the values obtained in Figure 2c and Figure 3b. Finally, if the temperature range is further narrowed, for example, even if only the temperature range of (180–190) K ((5.6–5.2) in 1000/K) is measured, then all deep levels are expressed. Therefore, we proved that a similar result as conventional CDLTS measurement could be obtained, even if the characteristic temperature $T_{peak}$ is confirmed through the I–V curve fitting error signal, and the LT–DLTS analysis is performed only in the minimum temperature range around $T_{peak}$. To further confirm that LT–DLTS can be performed in a very narrow temperature range, the ICTS analysis [21] was performed and confirmed that similar $E_{a}$ values were derived (Figure S7 of the SI). The additional effectiveness of our work is peak finding accuracy is better than the other comparable diagnostic tools like TSC. We compared this in Section S6 in Supplementary Information.

## 4. Conclusions

For a diagnostic signal for DLTS measurement, the current work proposed and demonstrated an I–V curve fitting error signal of the Schottky diode. From the I–V–T measurement of the Be-doped GaAs Schottky diode, by performing curve fitting in the forward region, a misfit error signal for temperature was obtained, and the shape of the error signal was confirmed to be very similar to the CDLTS signal and b1 signal. This is because the charged particle captured at the deep level in the characteristic temperature $T_{peak}$ emerges as an excess current in the I–V curve due to the emission phenomenon caused by thermal energy. This phenomenon is used to obtain the misfit errors between the experimental and theoretical values in the I–V curve fitting. Particularly we found that the results of LT–DLTS or ICTS analysis within a very small temperature range of (180–190) K based on the characteristic temperature $T_{peak}\;\approx$ 200 K, which showed the largest signal among error signals, show similar results as the CDLTS and FT–DLTS analysis analyzed in the wide temperature range of (80–400) K. In addition, we demonstrated that DLTS measurement could be achieved time-efficiently using the LT–DLTS or ICTS method with the defect characteristic temperature of the error signal. Our methodology is expected to open up new possibilities for effective defect analysis when evaluating the characteristics of devices, such as 2D materials.

## Data Availability

Not applicable.

## Supplementary Materials

The following supporting information can be downloaded at: https://www.mdpi.com/article/10.3390/nano13212866/s1. Refs. [21,28,38,39,40,41] are cited in Supplementary Materials.
