# Supplementary material for: Auxiliary Diagnostic Signal for Deep-Level Detection
Source: Nanomaterials (Basel). 2023 Oct 29;13(21):2866. doi: 10.3390/nano13212866 (PMC10648165; doi:10.3390/nano13212866)
Supplement: Supplementary file 1 [file nanomaterials-13-02866-s001.zip › nanomaterials-2644152-supplementary.pdf]

Supplementary Information

Auxiliary Diagnostic Signal for Deep-Level Detection

Il-Ho Ahn [1,*], Dong Jin Lee[1] and Deuk Young Kim [1,2]

1 Quantum-Functional Semiconductor Research Center, Dongguk University, Seoul 04620, Republic of Korea; jin514rin@naver.com (D.J.L.); dykim@dongguk.edu (D.Y.K.)
2 Division of Physics and Semiconductor Science, Dongguk University, Seoul 04620, Republic of Korea
* Correspondence: ihahn@dongguk.edu; Tel.: +82-2-2260-3951; Fax: +82-2-2260-3945

1. Structural characterization of the Be-doped p–GaAs thin film

Scanning electron microscopy (SEM) was employed to probe the sample's surface morphology and lateral structure. The SEM images shown in Fig. S1(a) and (b) display the smooth, mirror-like surface morphology (Fig. S1(a)). The highly epitaxial layer of Be-doped GaAs along the [311] direction has been achieved with a buffer layer (a thickness of 1μm) on GaAs (311)A substrate known to be useful for growing high-quality p-type materials as a substrate (Fig. S1(b)) [1]. Energy dispersive spectrometer (EDS) elemental mapping was carried out to access the Be distribution of the p-GaAs thin film. Fig. S2 clearly reveals the homogeneous distribution of all three elements (Bi, Ga and As) on the Be-doped GaAs thin film, and the atomic concentration of the Be ions in the GaAs host materials is approximately 8.5 at. %. In addition, the Hall effect measurement technique confirmed that the prepared Be-doped GaAs thin film was a p-type semiconductor with a hole concentration of 1.32×10^{17} cm^3 at 0.9 T, 300 K. Phase of Be doped p-GaAs epitaxial film was investigated by x-ray diffraction method, and the corresponding XRD pattern was shown in Fig. S3. Labeled crystalline planes

refer to files JCPDS 32-0389 [2]. Based on the XRD spectrum, the cubic zinc blende structure dominant with (311) plane and single crystalline is obtained with no peaks associated with the second phase.

2. Built-in potential of the p–GaAs Schottky diode

The built-in voltage V_{bi} in the p-type Schottky diode is expressed as follows [3]:

$$V_{bi} = \frac{1}{q}\big((\Phi_M - \chi\,) - (E_F - E_v)_{FB}\big) \tag{S1}$$

$$(E_F - E_v)_{FB} \approx \frac{E_g}{2} + kT\,ln\left(\frac{N_A}{n_i}\right) \tag{S2}$$

where for p–GaAs, we used $\Phi_M = 5.22$ eV, $\chi = 4.07$ eV, $N_A = 5 \times 10^{18}$ cm^{-3}, $n_i = 1.79 \times 10^{6}$ cm^{-3}, $E_g = 1.4$ eV, $T = 130$ K, and k is Boltzmann's constant.

We obtained the theoretical value V_{bi}= 0.11 V at 130 K representatively, which matches the experimental data in Fig. S4(a) well.

3. Reverse leakage current of the Schottky diode

The absolute reverse leakage current of the p–GaAs Schottky diode is about 0.85 mA at −5 V [see Fig. S4(a)]. A linear fit of the Arrhenius plot in Fig. S4(b) yields the thermal activation energy according to $I \propto \exp\left(-\frac{E_a}{kT}\right)$ [4]. The estimated thermal activation energy of 60 meV, which is exactly the same as one of the DLTS analysis results, can be attributed to the thermal excitation of holes from the deep trap state.

4. Simulation of CDLTS signal

To confirm CDLTS analysis data, we simulate the CDLTS signal with simple procedures. From the CDLTS analysis, we have three deep-level states with activation energies of (0.05, 0.2, and 0.3) eV. Therefore, we assume the multi-capacitance transient data are as below:

$$C(t,T) = \sum_{i=1}^{3} \Delta C_{0i} \exp\left(-e_p(T)t\right) \tag{S3}$$

$$e_p(T) = \sigma_p \gamma_p T^2 exp\left(-\frac{qE_a}{kT}\right) \tag{S4}$$

where, ΔC_{0i} is the coefficient obtained from experimental capacitance transient data (see Fig. S5(a)), γ_p = 1.7 × 10^{21} $cm^{-2}s^{-1}K^{-2}$ for p–GaAs, and E_a and σ_p are the activation energy and captured cross-section obtained from CDLTS analysis. To simulate the CDLTS signal, the DLTS signal of each temperature point T_k is calculated by the following formula:

$$\Delta C(t_2, t_1, T_k) = \Delta C_{0i} \exp\left(-e_p(T_k)t_2\right)) - \Delta C_{0i} \exp\left(-e_p(T_k)t_1\right)) \tag{S5}$$

Figure S6 shows that the simulated CDLTS signal coincides with those of the experimental DLTS signal at t_w = ${}^{1}/_{e_p}$ = 1.42 × 10^{-4} s in temperature position and peak heights. This means that the CDLTS analysis within the measured temperature range is reliable.

5. ICTS signal

To confirm that LT–DLTS can appear intensively in a minimal temperature range,

isothermal DLTS [5] was performed at three temperatures of (184, 186, and 188) K. As shown in Fig. S7(a), this also showed an ICTS signal with three peaks; using this, the E_a obtained from the Arrhenius plot was also confirmed to be consistent with the CDLTS, FT−DLTS, and LT−DLTS (Fig. S7(b)).

6. Comparison of effectiveness of error signal

To validate the effectiveness of our error signal, in Fig. S8, we compared our signal with the additional auxiliary thermally stimulated current (TSC) signal obtained from Fig.1(b). The TSC signal here is composed by picking up the current values for every temperature at a fixed voltage. The TSC signal below shows that the peaks under the forward and reverse regimes are less clear than our misfit error signals in Fig. 1(d).

Tables & Captions

Table SI. Physical mechanisms of electrical conduction of the p–GaAs Schottky diode. We obtained the current-transport components by adopting all the detailed fitting methods in Ref. [6].

Transport mechanism	Current model	
	Characteristic component	I–V model
Thermionic emission	I_{te0}	$I_{te} = I_{te0}\left[exp\left\{\frac{q(V-IR_s)}{k_BT}\right\}-1\right]$
Generation–recombination	I_{gr0}	$I_{gr} = I_{gr0}\left[exp\left\{\frac{q(V-IR_s)}{2k_BT}\right\}-1\right]$
Tunnelling	I_{tun0}	$I_{tun} = I_{tun0}\left[exp\left\{\frac{q(V-IR_s)}{E_0}\right\}-1\right]$
Leakage	-	$I_{lk} = \frac{V-IR_s}{R_t}$

Table SII. Comparison of accuracy and time-efficiency of available auxiliary tools for deep-level detection when using DLTS.

Available auxiliary tool	Auxiliary tool measurement Method	Measurement time	Peak finding accuracy	Peak finding method	DLTS methods applicable
Thermally stimulated current (TSC)	Ramp rate stimulated current	long	good	direct	CDLTS FT-DLTS LT-DLTS ICTS
Thermally stimulated capacitance (TSCAP)	Ramp rate stimulated capacitance	long	good	direct	
Error signal (Our work)	Temp scan I-V sweep	short	better	curve fitting	

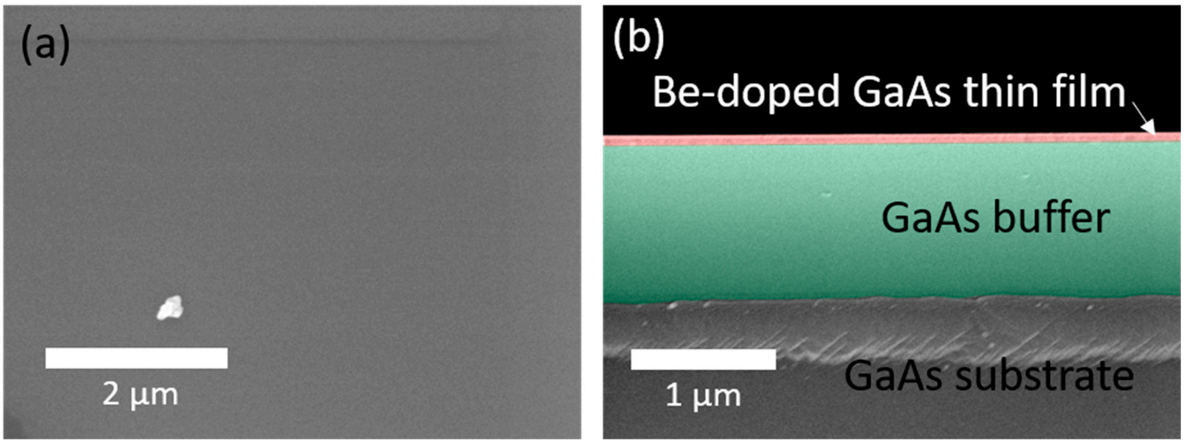

Figure S1. (a) Planar and (b) cross-sectional view of SEM images of Be-doped GaAs / GaAs buffer / GaAs substrate.

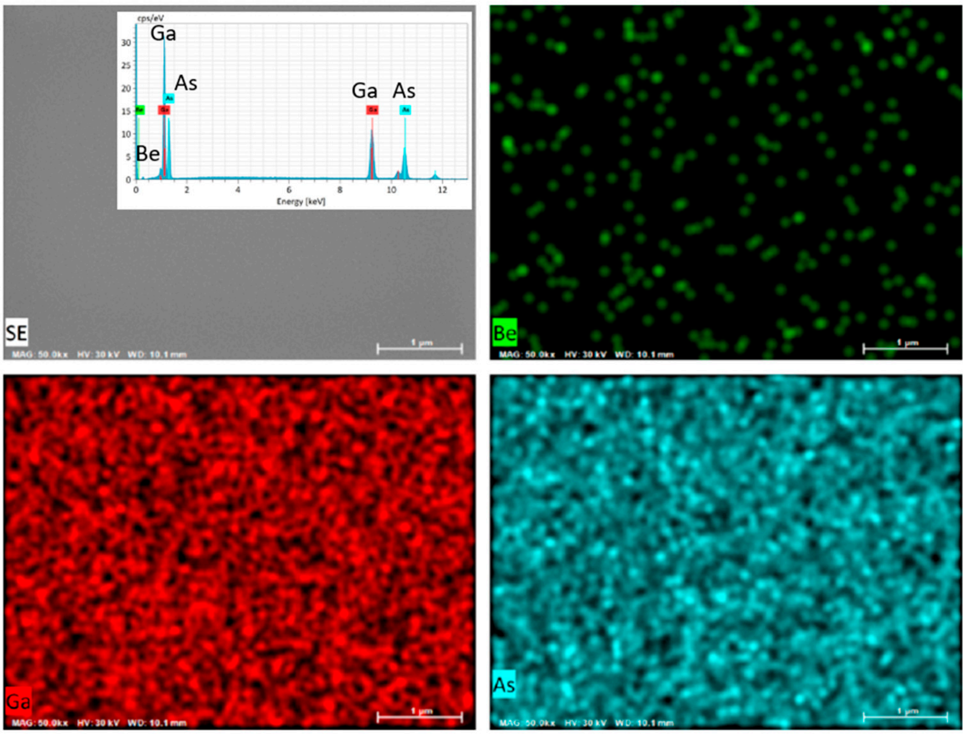

Figure S2. EDS elemental mapping of Be, Ga and As of the selectively Be-doped GaAs thin film region. The inset shows EDS spectra.

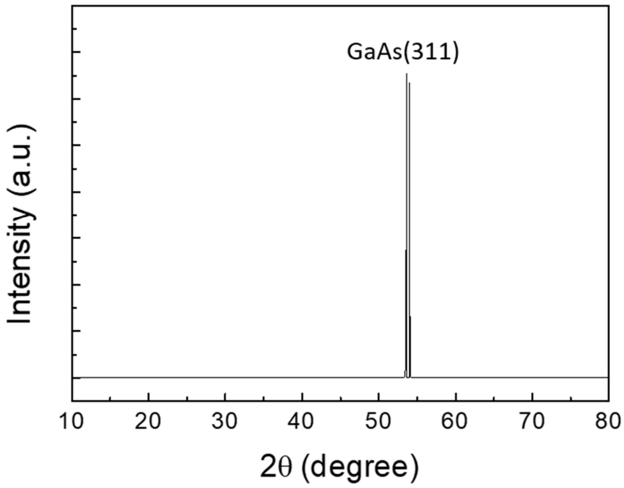

Figure S3. XRD diffraction curve obtained from Be-doped GaAs thin film.

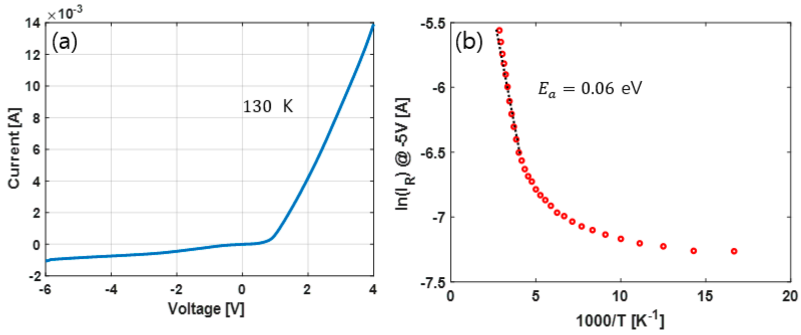

Figure S4. (a) I–V curve at 130 K. (b) Arrhenius plot of the reverse leakage current of the Schottky diode as a function of reciprocal temperature, measured at −5 V.

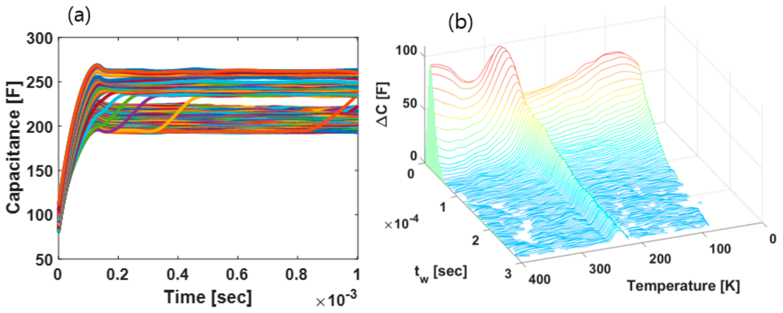

Figure S5. (a) Capacitance transient data for all temperature ranges, (80–400) K. (b) CDLTS signal at t_2/t_1 =2.

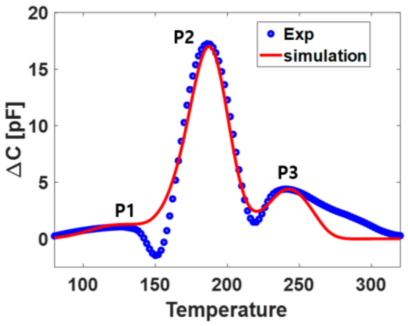

Figure S6. Simulation of CDLTS signal at t_2/t_1= 2, t_w = $^1/_{e_p}$ = 1.42 × 10^{-4} s.

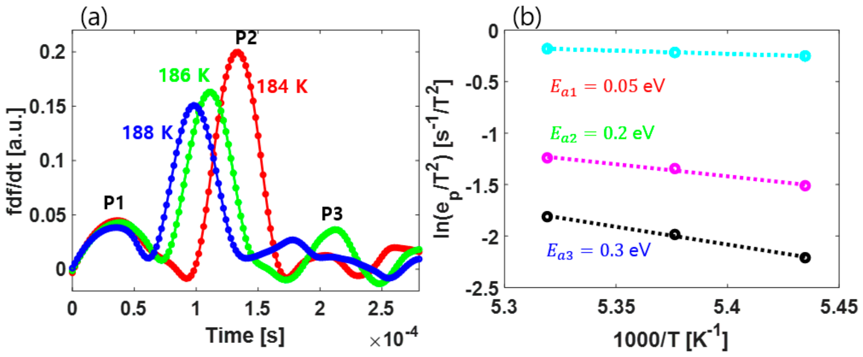

Figure S7. (a) Isothermal DLTS signal at (184, 186, and 188) K. (b) Arrhenius plot based on the ICTS signal in Fig. S7(a).

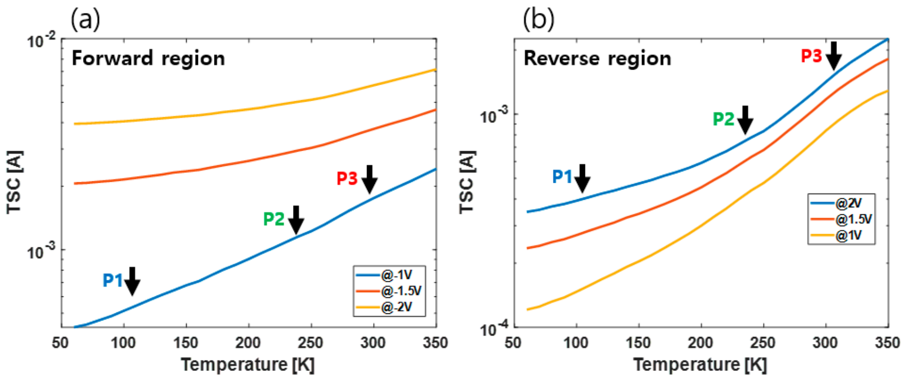

Figure S8. TSC signal under (a) the forward and (b) the reverse regime at given fixed bias voltages.

References

1. Olsson, L.Ö.; Björkqvist, M.; Kanski, J.; Ilver, L.; Nilsson, P.O. Surface electronic structure of GaAs(311)A studied by angle-resolved photoelectron spectroscopy. *Surface Science* **1996**, *366*, 121-128, doi:https://doi.org/10.1016/0039-6028(96)00658-9.
2. JCPDS (Joint Committee on Powder Diffraction Standards)/(ICDD) International Centre for Diffraction Data (2003) Powder diffraction file, Pennsylvania: JCPDS/ICDD. https://www.icdd.com/pdfsearch/.
3. Pierret, R. Semiconductor Device Fundamentals. **1996**, 483.
4. Peta, K.R.; Kim, M.D. Leakage current transport mechanism under reverse bias in Au/Ni/GaN Schottky barrier diode. *Superlattices and Microstructures* **2018**, *113*, 678-683,

doi:https://doi.org/10.1016/j.spmi.2017.11.056.
5. Okushi, H.; Tokumaru, Y. Isothermal Capacitance Transient Spectroscopy. *Japanese Journal of Applied Physics* **1981**, *20*, 261, doi:10.7567/JJAPS.20S1.261.
6. Suzue, K.; Mohammad, S.N.; Fan, Z.F.; Kim, W.; Aktas, O.; Botchkarev, A.E.; Morkoç, H. Electrical conduction in platinum–gallium nitride Schottky diodes. *Journal of Applied Physics* **1996**, *80*, 4467-4478, doi:10.1063/1.363408.
